# Effect of pharmacological manipulations on Arc function

**DOI:** 10.1016/j.crphar.2020.100013

**Published:** 2020-12-24

**Authors:** Dina W. Yakout, Nitheyaa Shree, Angela M. Mabb

**Affiliations:** Neuroscience Institute, Georgia State University, Atlanta, GA, United States

**Keywords:** Arc/Arg3.1, Addiction, Antidepressants, Antipsychotics, BDNF, Drugs of abuse, Inflammation

## Abstract

Activity-regulated cytoskeleton-associated protein (Arc) is a brain-enriched immediate early gene that regulates important mechanisms implicated in learning and memory. Arc levels are controlled through a balance of induction and degradation in an activity-dependent manner. Arc further undergoes multiple post-translational modifications that regulate its stability, localization and function. Recent studies demonstrate that these features of Arc can be pharmacologically manipulated. In this review, we discuss some of these compounds, with an emphasis on drugs of abuse and psychotropic drugs. We also discuss inflammatory states that regulate Arc.

## Highlights

• Arc is an immediate early gene whose expression is disrupted in neurological conditions.
• Arc expression in the brain can be modulated by drugs of abuse and psychotropic drugs.
• Arc might be a viable therapeutic target for neurological conditions.

## Graphical abstract

**Figure:**
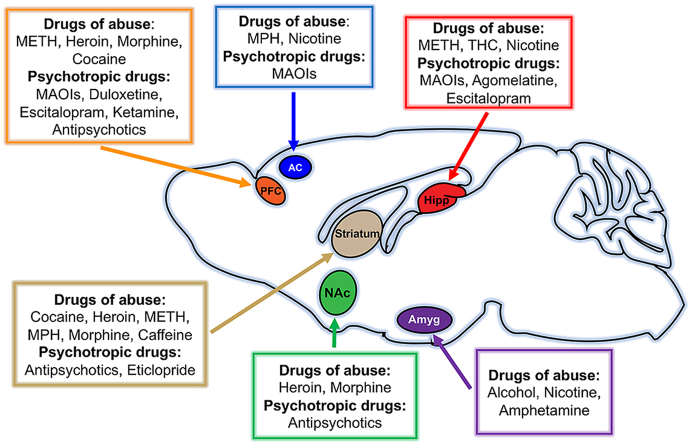

## 1 Introduction

Activity-regulated cytoskeleton-associated protein (Arc) is an immediate early gene (IEG) product whose levels rapidly increase and then decline as a response to changes in neural activity (Guzowski et al., 1999). Arc is elevated during memory acquisition, consolidation, and retrieval and is involved in the regulation of mood and reward-reinforcement (Li et al., 2015; Zhang and Bramham, 2020; Bramham et al., 2008; Ramirez-Amaya et al., 2005; Plath et al., 2006). Arc temporal dynamics are also critical for cognitive flexibility (Wall et al., 2018). Not surprisingly, Arc modulates Hebbian and non-Hebbian forms of synaptic plasticity including long-term potentiation (LTP) ([Bibr bib129][Bibr bib144], Plath et al., 2006), long-term depression (LTD) (Park et al., 2008; Waung et al., 2008), inverse synaptic tagging (Okuno et al., 2012), and homeostatic plasticity (Shepherd et al., 2006; Mabb and Ehlers, 2018). Changes in Arc expression in the prelimbic, infralimbic, anterior cingulate, prefrontal cortex, and amygdala have also been shown to play a role in mood regulation (Li et al., 2015). Arc regulation in the mesocorticolimbic system, including the nucleus accumbens (NAc), medial prefrontal cortex (mPFC), and dorsomedial striatum modulates the reward and reinforcement system and affects drug-seeking behaviors (Li et al., 2013). Modulation of Arc has further been implicated in several neurological disorders including Alzheimer's disease (AD), schizophrenia, depression, autism, Angelman syndrome, and Fragile X syndrome (Gallo et al., 2018; Wilkerson et al., 2018).

Given Arc's involvement in neurological and neuropsychiatric conditions, along with recent findings for its role in drug seeking behavior, it is viable to propose that Arc modulation at the transcriptional, translational and posttranslational levels may be a relevant therapeutic target. Here, we discuss how Arc can be pharmacologically manipulated, with a specific focus on neuropsychotropic and inflammatory pathways that modulate Arc in key brain regions that are involved in addiction, reinforcement and reward.

## 2 Arc structure

To establish Arc as a potential clinical target, it is important to discuss recent findings regarding its genetic organization and protein structure. The mouse *Arc* promotor has a major synaptic activity-responsive element (SARE) located >5 ​kb upstream of its transcription initiation site, which is necessary and sufficient to replicate endogenous *Arc* transcription. The promoter consists of a cluster of neuronal activity-dependent *cis*-regulatory elements of closely localized binding sites for cAMP-response element binding protein (CREB), myocyte enhancer factor 2 (MEF2) and serum response factor (SRF). This SARE falls within a region that is highly conserved in humans (Kawashima et al., 2009). Based on this discovery, an enhanced synthetic activity-regulated promotor was generated (E-SARE) with a more than 20-fold higher expression and 30-fold higher induction ratio than the *c-fos* promoter. This E-SARE was used in mice to map active neuronal ensembles in multiple brain regions (Kawashima et al., 2013). SRF, a transcription factor that is regulated by synaptic activity (Knoll and Nordheim, 2009), binds to the *Arc* SARE at two locations that are approximately 1.1 and 6.9 ​kb upstream from the translation start site. SRF binding at location 6.9 is required for the late phase of LTD in mouse cultured cerebellar Purkinje cells (Smith-Hicks et al., 2010). The rapid induction of *Arc* is due to the phenomenon of “promotor proximal Poll II stalling” that is mediated by negative elongation factor (NELF), where RNA polymerase II (Pol II) is recruited to the transcription start site (TSS) and stalls in the proximity of the promotor until heightened neuronal activity releases the stalled Pol II (Saha et al., 2011).

*Arc* mRNA is unique among other IEGs in that newly transcribed *Arc* is rapidly localized to dendrites, selectively in domains contacted by active synapses (Lyford et al., 1995; Link et al., 1995). Constructs that contain the 3′ untranslated terminal region (UTR) of *Arc* localize with high precision to a small domain at the base of the dendritic spine even when the transcript is tethered to a different protein product suggesting that the 3’ UTR of *Arc* is sufficient for its dendritic localization. Additionally, translation inhibitors do not interfere with *Arc* mRNA localization, indicating that targeting of *Arc* is a function of its mRNA rather than its protein (Dynes and Steward, 2007, 2012; Steward et al., 1998). Similarly, *Arc* mRNA degradation is activity-dependent, where both localization and degradation appear to rely on N-methyl D-aspartate receptor (NMDAR) activation (Farris et al., 2014). *Arc* also accumulates at inactive synapses through an interaction with calmodulin-dependent kinase II (CaMKII) beta (Okuno et al., 2012). Both paired pulse and metabotropic glutamate receptor (mGluR)-dependent LTD rapidly induce *Arc* translation (Park et al., 2008).

Structurally, Arc is composed of two lobes; the N-lobe and the C-lobe, which together form the GAG domain, sharing similarities to capsid-forming Gag proteins (Zhang et al., 2015; Pastuzyn et al., 2018). The N-lobe contains a matrix domain and forms a hydrophobic pocket, allowing it to selectively bind to synaptic scaffolding proteins such as CaMKII and the transmembrane AMPA (α-amino-3-hydroxy-5-methyl-4-isoxazolepropionic acid) receptor regulatory protein, TARPγ2 (also known as Stargazin) (Zhang et al., 2015). The acidic C-lobe of Arc (amino acids 280–396 in rat) contains a capsid-like domain with an adjacent disordered C-terminal tail (Boldridge et al., 2020; Nielsen et al., 2019). Given this domain organization, Arc has been shown to assemble into viral-like capsids that are proposed to form via electrostatic interactions of the C-lobes (Pastuzyn et al., 2018; Boldridge et al., 2020; Nielsen et al., 2019). A recent study demonstrated that a 28-amino acid helical coil motif in the N-terminal region is necessary and sufficient for Arc to self-assemble into viral-like capsids (Eriksen et al., 2020). Arc can exist as a monomer but can also undergo self-oligomerization, which can be reversible depending on external conditions. In high ionic environments, Arc oligomerizes, leading to stabilization of its C-lobe (amino acids 209–365 in human) (Myrum et al., 2015). These structural features allow Arc to directly interact with endogenous binding partners that control synaptic function like TARPγ2, NMDA receptor subunits, and CaMKII (Zhang et al., 2015; Nielsen et al., 2019; Jackson and Nicoll, 2011).

Arc also undergoes various post-translational modifications (PTMs) including ubiquitination, SUMOylation, phosphorylation, acetylation, and palmitoylation (Mabb and Ehlers, 2018; Carmichael and Henley, 2018). These PTMs play important roles in Arc functions that include oligomerization, proteasome-dependent degradation, AMPA receptor trafficking, association with the cytoskeleton, consolidation of LTP, mGluR-LTD, and subcellular localization (Greer et al., 2010; Craig et al., 2012; Nair et al., 2017; Nikolaienko et al., 2017; Mabb et al., 2014; Zhang et al., 2019; Gozdz et al., 2017; Barylko et al., 2018; Lalonde et al., 2017). For example, phosphorylation within the GAG domain of Arc by CaMKIIα regulates its oligomerization (Zhang et al., 2019). Deacetylation of Arc by a protein lysine deacetylase, or phosphorylation of Arc by GSK3α and GSK3β, promotes Arc degradation (Gozdz et al., 2017; Lalonde et al., 2017).

## 3 Drugs of abuse and Arc

Several studies have demonstrated an increase in *Arc* mRNA in the striatum and various other brain areas in response to amphetamine and cocaine (Tan et al., 2000; Fosnaugh et al., 1995; Fumagalli et al., 2006; Kodama et al., 1998). *Arc* is increased in the rat striatum within 15 ​min of cocaine administration and returns to basal levels by 2 ​h. This effect is suppressed by pretreatment with reserpine, which depletes catecholamines and dopamine (DA) stores at 24 and 3 ​h before cocaine treatment. Suppression is also observed following intrastriatal injections of the selective DA neurotoxin 6-hydroxydopamine (6-OHDA) or the DA receptor D1 antagonist SCH- 23390. Interestingly, DA D2 antagonists such as haloperidol, spiperone and eticlopride also induce *Arc* in the striatum. Collectively, these findings demonstrate a role for DA D1 antagonists in inducing *Arc* in response to cocaine (Fosnaugh et al., 1995; Fumagalli et al., 2006). Similarly, within 1 ​h of a single methamphetamine (METH) intraperitoneal injection, *Arc* significantly increases in the parietal, orbital and medial prefrontal cortex (mPFC) and to a lesser degree in medium-sized striatal neurons and the hippocampus, returning to basal levels by 6 ​h. Pretreatment with SCH- 23390 or the NMDA receptor antagonist MK-801 attenuates these effects suggesting a synergistic role for D1 and NMDA receptors in *Arc* modulation. However, a repeated METH regimen does not affect basal *Arc* (Kodama et al., 1998; Fujiyama et al., 2003) suggesting a potential role for Arc in synaptic tuning associated with an acute response to METH. The METH-induced increase in *Arc* in the cerebral cortex suggests that it may also play a role in the induction and maintenance of behavioral sensitization due to METH (Kodama et al., 1998). Interestingly, pretreatment with an intrastriatal infusion of the *mu* opioid receptor antagonist D-Phe-Cys-Tyr-D-Trp-Arg-Thr-Pen-Thr-Pen-Thr-NH2 (CTAP) attenuates the METH-induced increase in *Arc* (Horner et al., 2010) suggesting that opioid signaling can modulate dopaminergic responses to METH in the striatum.

Recreational drugs such as 3,4-methylenedioxymethamphetamine (MDMA/ecstasy) and delta-9-tetrahydrocannabinol (THC/Cannabis) are commonly abused by adolescents (Malone et al., 2010). MDMA increases serotonin release and inhibits its reuptake, while THC activates CB1 cannabinoid receptors (Cunningham et al., 2009; Rodriguez de Fonseca et al., 1993). Rats treated with MDMA and THC in adolescence (postnatal day 28–45) show long-term decreases in Arc that persist into adulthood in the cortex and the hippocampus in a sex-dependent manner. THC reduces Arc in the hippocampus of both males and females, while reductions in Arc in the PFC are observed only in females. MDMA also selectively reduces Arc in the female hippocampus (Llorente-Berzal et al., 2013).

*Arc* is upregulated in the mPFC at 1 day of abstinence in heroin self-administrating rats compared to rats yoked to heroin, but these changes do not persist following 14 days, which suggests that Arc might be involved in establishing the memory of the self-administration behavior (Kuntz et al., 2008). Runway training with heroin, in which rats are trained to run to a goal area to self-administer the drug, significantly increases Arc in the mPFC, the nucleus accumbens (NAc) and the striatum, with differences in the temporal expression of Arc between the dorsomedial and ventrolateral striatum, suggesting the involvement of Arc in goal-directed behavior in the ventrolateral striatum. SCH2 3390 and MK-801 abolish this increase and suppress drug seeking behavior (Li et al., 2013).

Withdrawal from psychostimulants such as amphetamines produce manic and depressive symptoms (Mamelak, 1978). Acute _D_-amphetamine challenge significantly increases *Arc* in the frontal cortex, visual cortex, CA1 and amygdala (Pathak et al., 2015). Moreover, *Arc* mRNA significantly increases in the insular frontal cortex, visual cortex, entorhinal cortex, CA3, CA1, subiculum and striatum on day 17 of amphetamine withdrawal (Pathak et al., 2015). This withdrawl-induced increase is associated with a decrease in functional connectivity between frontal cortex and striatum and an increase in functional connectivity between the amygdala and the hippocampus. The observed changes in connectivity are coupled to behavioral signs of amphetamine sensitization which include hyperactive locomotor responses to restraint stress and amphetamine challenge, reduction in sucrose consumption, impaired memory consolidation and impaired nest building (Pathak et al., 2015). In prepubertal rats, *Arc* is increased in the striatum and cingulate cortex in response to daily treatment of methylphenidate (MPH) for 14 days and is also induced with a challenge MPH dose following a 4-week drug-free period (Chase et al., 2007). An increase in *Arc* in the PFC is also observed following cocaine self-administration training (Fumagalli et al., 2009a) suggesting a role for Arc in drug-related learning. Increases in *Arc* are also found in *c-fos* positive PFC neurons following cue-induced heroin seeking (Fanous et al., 2013). Infusion of *Arc* antisense oligodeoxynucleotides into the striatum attenuates extinction of cocaine-seeking (Hearing et al., 2011), suggesting that *Arc* induction in the striatum is required for drug-seeking behaviors. Recently, Penrod et al. described a role for Arc in volitional cocaine-taking intravenous self-administration (IVSA). They found that despite *Arc* Knock-out (KO) mice showing deficits in fear conditioning, they have intact contextual and reward learning along with normal appetitive classical and operant conditioning. However, *Arc* KO mice have a flat dose-response curve, that unlike WT mice, continue to self-administer cocaine at all doses, suggesting a decrease in the hedonic set-point and in the reinforcing effects of the drug (Penrod et al., 2020). The consequences of selective Arc disruption in the PFC on cocaine self-administration have yet to be established.

Arc is implicated in the reinstatement of drug seeking behavior. An increase in *Arc* is observed in subregions of the PFC upon re-exposure to cocaine-related cues (Zavala et al., 2008). In rats, cocaine-related context-induced relapse to drug seeking increases *Arc* in the mPFC, orbitofrontal cortex (OFC), striatum, NAc, basolateral amygdala (BLA) and dorsal hippocampus (Hearing et al., 2008a, 2008b, 2010). Visual cue-elicited reinstatement of cocaine seeking is also associated with increases in *Arc* in the mPFC and BLA, while priming-induced reinstatement causes a more widespread *Arc* induction in cortical regions (especially the anterior cingulate and motor cortex) (Ziolkowska et al., 2011). These findings suggest a unique brain-specific pattern of Arc induction in different forms of drug relapse that include drug-paired stimulus-induced relapse (cue) and drug slip/lapse (priming) (Bossert et al., 2013; Beardsley and Shelton, 2012).

Following chronic morphine treatment, Arc mRNA and protein are increased in the rat striatum (Marie-Claire et al., 2004). Upon naloxone-precipitated withdrawal from morphine, *Arc* remains elevated in the frontal cortex, suggesting that persistent changes during opiate dependence may be due to a permanence in synaptic remodeling (Ammon et al., 2003). Additionally, *Arc* is induced upon morphine withdrawal in the central and basolateral amygdala along with the basolateral amygdala during reactivation of opiate withdrawal memories by re-exposure to the withdrawal-paired environment (Lucas et al., 2008). In the NAc, Arc protein significantly increases in the core after 2 ​h of morphine treatment, while it increases in the shell but not the core after context-induced drug seeking. Arc in the NAc is also essential for morphine-related addiction memory. Knockdown of Arc in the core blocks the acquisition and expression of morphine conditioned-place preference (CPP) and reinstatement, while knockdown in the shell only impairs the expression of morphine CPP (Lv et al., 2011). In a recent study, activation of neural activity patterns in the striatum was measured using cellular compartment analysis of temporal activity by fluorescence *in-situ* hybridization (catFISH) for *Arc* and *Homer1a*. Although separate injections of cocaine and heroin induce *Arc* and *Homer1a* with a similar time course, injection of cocaine followed by heroin 25 ​min later engages distinct patterns of neurons in the striatum, providing supporting evidence that these two drugs operate through different mechanisms in the brain and encode distinct representations in the striatum (Vassilev et al., 2020).

Arc is also modulated in models of alcoholism, where acute ethanol exposure through intraperitoneal injections in rats increases Arc protein and dendritic spine density in the central and medial amygdala, while withdrawal after long term exposure is associated with a decrease in Arc and dendritic spine density in these same areas. Changes in brain derived neurotrophic factor (BDNF) and its receptor tyrosine Kinase B (trkB) mirror the changes in Arc. Additionally, infusion of BDNF into the central amygdala normalizes Arc levels and attenuates the onset of the withdrawal-related anxiety, suggesting that BDNF-Arc signaling in the central and medial amygdala is involved in alcohol dependence and anxiety related to drinking behavior (Pandey et al., 2008).

Intraperitoneal injections of caffeine, an adenosine (A) receptor antagonist, or the A_1_ antagonist 1,3-dipropyl-8 cyclopentylxanthine (DPCPX) induce Arc mRNA and protein in striatonigral and striatopallidal neurons. This effect appears to be under the control of the dopaminergic system, where pretreatment with the D2/D3 agonist quinpirole almost completely blocks this caffeine-induced increase in *Arc*, while the D1 antagonist SCH2 3390 attenuates it in a dose-dependent manner (Dassesse et al., 1999). In juvenile rats, acute nicotine exposure by intraperitoneal injections at postnatal day (P) 5,7 and 10 lead to an increase in *Arc* in cingulate cortex, hippocampus, caudate and central amygdala, while *Arc* increases in the bed nucleus of stria terminalis after acute exposure to nicotine at P10 only, indicating that age is an important factor in nicotine-induced regulation of *Arc* (Schmitt et al., 2008).

## 4 Psychotropic drugs

Arc is affected by serotonin (5-HT) in various brain areas. While the 5-HT precursor L-tryptophan by itself fails to induce an increase in *Arc*, its combined intraperitoneal injection with the monoamine oxidase inhibitor (MAOI), tranylcypromine increases *Arc* in the orbital, frontal and parietal cortices the striatum and decreases in the CA1 region of the hippocampus. The 5-HT releasing agent p-chloroamphetamine and the 5-HT_2_ agonist 1-(2,5-dimethoxy-4-iodophenyl)-2-aminopropane (DOI) also increase *Arc* in cortical and striatal areas. 5-HT depletion using the tryptophan hydroxylase inhibitor *p*-chlorophenylalanine and pretreatment with the 5-HT_2_ antagonist ketanserin attenuate increases in the cortices and striatum, with no effect on the reduction observed in CA1. This suggests that *Arc* is regulated by endogenous 5-HT release in the cortex and striatum, where 5-HT_2_ appears to play an important role in the cortex (Pei et al., 2000). Several drugs that manipulate 5-HT are used to treat mood disorders and need several weeks to achieve their therapeutic effect (Machado-Vieira et al., 2010). Consistent with this, twice-daily injections for 14 days with the MAOIs paroxetine, desipramine and venlafaxine increase *Arc* in the parietal, frontal, orbital, cingulate cortex and CA1 of the hippocampus, with no changes detected in the caudate putamen or the dentate gyrus, while tranylcypromine increases *Arc* in the dentate gyrus. However, acute administration of these drugs has no effect on *Arc*, possibly because acute administration of antidepressant drugs causes a compensatory decrease in 5-HT due to feedback regulatory mechanisms involving 5-HT_1A_ receptors (Blier and Ward, 2003), although tranylcypromine does cause an increase in *Arc* in the parietal cortex (Pei et al., 2003). S35966A, a dual α_2_-adrenoceptor antagonist and 5-HT-noradrenaline reuptake inhibitor more potently increases *Arc* compared to the selective serotonin and norepinephrine reuptake inhibitor (SSNRI) venlafaxine in the cortical and hippocampal areas following acute treatment (Serres et al., 2012). Duloxetine, also an SSNRI, induces *Arc* primarily in the frontal cortex after chronic treatment suggesting a role for Arc in long-term adaptive changes with chronic antidepressant treatment (Molteni et al., 2008).

Selective 5-HT_1A_ receptor antagonists such as WAY 100635 and NDA-299 combined with the selective serotonin reuptake inhibitor (SSRI) paroxetine also increase *Arc* in frontal, parietal and piriform cortices and the caudate putamen, while none of these drugs have an effect on *Arc* when administered without paroxetine, suggesting the involvement of other 5-HT receptor subtypes (Tordera et al., 2003; Castro et al., 2003). Agomelatine, an MT_1_/MT_2_ melatonergic agonist and the 5-HT_2c_ receptor antagonist, also upregulates *Arc* in the hippocampus (Calabrese et al., 2011).

Interestingly, electroconvulsive stimulation (ECS), which remains one of the most effective treatments against severe depression (Berton and Nestler, 2006), strongly induces *Arc* at 1 and 4 ​h post exposure (Lyford et al., 1995), and causes a significant increase in DNA methylation of the *Arc* gene promoter at 24 ​h suggesting repression of *Arc* expression for a long period post-ECS (Dyrvig et al., 2012). A genetic rat model of depression called Flinders sensitive line, displays depression-like behaviors accompanied by reduction of *Arc* in the prefrontal cortex and hippocampus as well as reduction in mitogen-activated protein kinase (MEK) activity. Chronic treatment with the SSRI escitalopram as well as increasing 5-HT postsynaptic function via stimulation of the cAMP/mitogen-activated protein kinase (MAPK) signaling cascades restores some cognitive functions such as memory performance and *Arc*, suggesting a role for the 5-HT/MEK cascade in regulating *Arc* in depression (Eriksson et al., 2012).

Unlike other antidepressants, the non-selective NMDA receptor antagonist ketamine produces antidepressant effects within hours of its administration that last up to 7 days (Berman et al., 2000). In rat models of depression, a low dose of 10 ​mg/kg ketamine activates the mTOR signaling pathway in the PFC, leading to an increase in synaptic proteins that peak at 2–6 ​h and remain elevated up to 72 ​h, with Arc protein showing a more transient increase. The selective NR2B antagonist Ro 25-6981, which also activates the mTOR pathway, causes a more rapid and transient induction of Arc at 1 ​h that returns to normal by 6 ​h, and produces rapid antidepressant effects in the forced swim and novelty-suppressed feeding tests (Li et al., 2010).

Both typical and atypical antipsychotic drugs block D2 receptors (Rampino et al., 2018) and have been shown to modulate Arc (Bruins Slot et al., 2009; de Bartolomeis et al., 2013). The typical (first generation) antipsychotic haloperidol increases *Arc* in the NAc and striatum at 1 ​h post treatment, while the atypical (second generation) drug clozapine significantly reduces *Arc* at 6 ​h in the thalamus and hypothalamus (Robbins et al., 2008). Another study compared haloperidol to a different atypical drug, olanzapine. A single injection of haloperidol induces *Arc* in the striatum at 30, 60, and 120 ​min, while olanzapine induces *Arc* only at the 30 ​min time point. In contrast, both drugs cause a sharp decline in *Arc* in the frontal cortex at 60 ​min. The atypical D2 antagonist raclopride produces similar effects to haloperidol and olanzapine, inducing *Arc* in the striatum and reducing it in the frontal cortex, while the D2 agonist quinpirole (which has lower affinity to D2 than olanzapine) produces no change in *Arc* (Fumagalli et al., 2009b). One study compared the effect of acute and chronic treatment (once daily for 21 days and 24 ​h following withdrawal of haloperidol and clozapine) in rats on Arc protein. In the caudate nucleus, Arc increases following acute and chronic treatment with haloperidol, while clozapine has no effect. In the NAc, haloperidol increases Arc following acute treatment but decreases it following chronic treatment, while clozapine increases Arc in the NAc shell but not the core following acute treatment. In the mPFC and cingulate cortex, only clozapine has an effect where it decreases Arc following acute and chronic administration. Amisulpride, a benzamide which is known for fewer motor side effects compared to haloperidol despite being a high-affinity D2/D3 receptor blocker (Grunder et al., 2009), has a region-selective effect on *Arc* in the striatum and induces significantly lower levels of *Arc* than those produced by haloperidol that is restricted to the medial striatum (de Bartolomeis et al., 2013). Interestingly, the first-generation anti-psychotics thioridazine and trifluoperazine have been shown to inhibit the Arc N-lobe binding to TARPγ2 through mechanisms independent of their action on D2 and their antipsychotic function (Zhang et al., 2015).

Several studies point out that schizophrenia patients consume increased amounts of nicotine and caffeine (Williams and Gandhi, 2008). Coffee drinking and tobacco smoking can modulate the haloperidol-induced changes in Arc mRNA and protein expression. One study showed that cotreatment of rats with caffeine and haloperidol reduces the increase in Arc in the striatum compared to treatment with haloperidol alone, while cotreatment with nicotine and haloperidol produces significantly higher Arc in the cortex compared to haloperidol alone (de Bartolomeis et al., 2018). Phencyclidine (PCP), an NMDA antagonist with psychomimetic effects that resemble positive and negative symptoms of schizophrenia, produces opposite effects on *Arc* mRNA in juvenile and adult rats in the mPFC, orbitofrontal cortex and NAc shell, where it decreases expression in juveniles and increases it in adults when administered for 5 days (Thomsen et al., 2010). Pretreatment with atypical antipsychotics 1 ​h before administration of PCP in adult rats significantly reduces the PCP-induced increase in *Arc* in the mPFC and NAc suggesting that Arc modulation could be a key factor in the therapeutic properties of these drugs (Nakahara et al., 2000).

## 5 Brain derived neurotrophic factor (BDNF) and Arc

BDNF is a neurotrophic factor that regulates the differentiation and survival of neurons through activation of tyrosine receptor kinase B (TrkB) (Haapasalo et al., 2002). Infusion of BDNF into the dentate gyrus of rats increases *Arc* mRNA in the granular and molecular layers at 2 ​h, with a three-fold increase in Arc protein at 3 ​h. This effect is abolished with the MEK inhibitor U0126 (Ying et al., 2002). The BDNF-induced increase in Arc in synaptoneurosomes is also partially inhibited by pretreatment of the tyrosine kinase non-specific inhibitor K252-alpha, and to a greater extent with the NMDA antagonist MK801 suggesting a role for NMDA receptors (Yin et al., 2002). BDNF also induces *Arc* in rat neuronal cultures and organotypic slices, an effect that can be potentiated by inhibiting AMPARs, which negatively regulate *Arc* transcription (Rao et al., 2006). One study demonstrated the importance of the serum-response element 1 (SRE1) of the *Arc* gene regulatory region in modulating the BDNF-induced *Arc* response, where SRE1 binds SRF in an activity-dependent manner to regulate *Arc* transcription (Pintchovski et al., 2009). Arc is necessary for BDNF-induced LTP induction and consolidation, where infusion of *Arc* antisense into the medial perforant path before BDNF infusion blocks the induction of BDNF-LTP and causes rapid reversal of ongoing BDNF-LTP when infused 2 ​h after BDNF (Messaoudi et al., 2007). BDNF also plays an important role in adult neurogenesis in the hippocampus (Scharfman et al., 2005) and infusion of *Arc* antisense into the rat dentate gyrus blocks the pro-neurogenic effects of BDNF (Kuipers et al., 2016).

## 6 Interfering with Arc removal

As discussed above, Arc PTMs play an important role in regulating the dynamics of Arc turnover. The proteasome inhibitor MG-132 interferes with removal of Arc by the ubiquitin proteasome system (UPS) (Mabb et al., 2014; Rao et al., 2006). Mutations of Arc ubiquitination sites (ArcKR) interfere with its proteasome-dependent removal, leading to abnormal elevations in Arc and deficits in cognitive flexibility (Wall et al., 2018). The glycogen synthase kinases α and β (GSK3α/β) are serine/threonine kinases that catalyze Arc phosphorylation and degradation. The GSK3α/β inhibitor CHIR 98014 (CH98) augments the effect of NMDA on Arc and slows down Arc degradation (Gozdz et al., 2017). Protein lysine deacetylase (KDAC) inhibitors, such as AK-7 and oxamflatin can increase Arc by limiting its degradation (Lalonde et al., 2017). A reduction in autophagy and accumulation of ubiquitinated Arc is observed in Fragile X syndrome model mice. Inhibiting autophagosome formation by addition of 3-methyladenine (3-MA), and/or the lysosomal inhibitor leupeptin in combination with ammonium chloride inhibits the autophagy pathway and leads to Arc accumulation in hippocampal neurons. Enhancing autophagy by removing the inhibitory autophagy signaling protein Raptor using shRNA corrects excessive Arc and rescues synaptic and cognitive deficits in Fragile X syndrome model mice (Yan et al., 2018).

## 7 Stress, the immune system and Arc

Arc has recently been shown to play a role in the inflammatory response inside and outside the central nervous system (Rosi, 2011). Neuroinflammation alters patterns of *Arc* in the brain. In rats, chronic infusion with lipopolysaccharide (LPS) into the fourth ventricle for 28 days results in elevated exploration-induced *Arc* and an increase in the percentage of neurons expressing *Arc* in the dentate gyrus and CA3 regions, which is accompanied by activation of microglia as revealed by OX-6 immunoreactivity. However, LPS-induced inflammation does not alter basal *Arc* nor affect Arc translation and removal (Rosi et al., 2005). Interestingly, memantine, the NMDA receptor antagonist widely used to manage AD, prevents these alterations when infused with LPS at concentrations that produce similar plasma levels to those produced by therapeutic doses in humans, and results in significant improvement in acquisition and retention in the water maze, suggesting these alterations are mediated through NMDA receptors (Rosi et al., 2006). *Arc* catFISH analysis demonstrates unstable CA3 pyramidal neuron activity between two exposures to the same contextual environments during LPS-induced chronic neuroinflammation, an effect that is partially normalized by memantine (Rosi et al., 2009). Conversely, mice receiving whole brain irradiation, similar to therapeutic irradiation commonly used to treat brain tumors, show a reduction in the percentage of neurons expressing Arc mRNA and protein, which is associated with a significant increase in activated microglia at 2 months post-irradiation (Rosi et al., 2008).

While Arc has been conventionally studied in the context of its expression and function in neurons, it has recently come into the limelight as a mediator outside of the nervous system. Arc plays an important role in regulating peripheral cellular stress. Exposure of cells to toxic chemicals or oxidative stress launches the cellular defense mechanism known as the heat shock response (HSR), which is characterized by the induction of heat shock proteins (Hsps) (Neef et al., 2011). Diamide, the disulfide crosslinker and sodium arsenite, which is used as a pesticide and causes protein misfolding, induces Arc transiently in thermotolerant HeLa cells where it is rapidly degraded by the ubiquitin proteasome system (UPS) with a half-life of approximately 30 ​min. Arc then inhibits the activation of heat shock factor 1 (HSF1) leading to a decrease in the expression of Hsp27 and Hsp70, suggesting that Arc is part of a feedback loop to regulate the HSR (Park et al., 2019).

Arc is also a mediator of inflammatory migratory dendritic cell (migDC) migration from the skin to draining lymph nodes for inflammation-mediated T cell activation. Arc is enriched in four different subsets of migDC as well as Langerhans cells in the skin and draining lymph nodes (Tintelnot et al., 2019). The functional sphingosine 1-phosphate receptor antagonist fingolimod (FTY720), which impairs migDC influx into lymph nodes and is used in the treatment of multiple sclerosis, reduces *Arc* mRNA in migDC and in draining lymph nodes (Ufer et al., 2016). A recent preprint showed a new role for Arc in mediating neuroinflammatory responses in the skin, where Arc increases in dorsal root ganglion neurons in response to the inflammatory mediators neurotrophic growth factor (NGF) and interleukin-6 (IL-6), accumulating in the skin. Arc null mice exhibit an exaggerated inflammatory response that is reversed by addition of extracellular vesicles (exosomes) containing Arc (Barragan-Iglesias et al., 2020).

## 8 Conclusion

Functions of Arc are tightly related to glutamatergic transmission via NMDARs (Balu and Coyle, 2014), AMPARs (Chowdhury et al., 2006) and metabotropic glutamate receptor 5 (mGluR5s) (Kumar et al., 2012). The serotonergic system also induces Arc through mechanisms that involve both 5-HT_1A_ and 5-HT_2A_ receptors (Pei et al., 2003; Tordera et al., 2003). Additionally, Arc is regulated by the dopaminergic system, particularly in the striatum and the NAc, brain regions that play an important role in reward and reinforcement. Several drugs of abuse alter Arc in these regions suggesting a role for Arc in drug-induced learning, drug-seeking behavior and drug withdrawal (Pathak et al., 2015; Zavala et al., 2008) (Table 1).

**Table 1:** List of drugs of abuse, clinical names, general mechanism of action, and effect on Arc.

Drugs of Abuse				
Drug	Clinical/Trade Name	General Mechanism	Effect on Arc	Reference
alcohol		binds to ACh, 5-HT, GABA, and NMDA receptors; mainly increases effects of GABA	acute exposure increases Arc in central and medial amygdala; withdrawal after long-term exposure decreases Arc in central and medial amygdala	Pandey et al. (2008)
amphetamine	Adderall, Dexedrine	increases release of dopamine, NE, 5-HT from presynaptic terminal	acute administration increases *Arc* in frontal cortex, visual cortex, CA1 hippocampus, and amygdala; withdrawal increases *Arc* in insular frontal cortex, visual cortex, entorhinal cortex, CA3 hippocampus, CA1 hippocampus, subiculum, and striatum	(Tan et al., 2000; Pathak et al., 2015)
caffeine		adenosine receptor antagonist	increases *Arc* and Arc in striatonigral and striatopallidal neurons	Dassesse et al. (1999)
cocaine		blocks reuptake of dopamine, NE, 5-HT	increases *Arc* in striatum, PFC; relapse or cue-induced reinstatement increases *Arc* in medial PFC, OFC, striatum, NAc, BLA, dHP, anterior cingulate, and motor cortex	(Fosnaugh et al., 1995; Fumagalli et al., 2006, 2009a; Zavala et al., 2008; Hearing et al., 2008a, 2008b, 2010; Ziolkowska et al., 2011)
heroin		opioid receptor agonist	increases Arc in mPFC, NAc, and striatum	(Kuntz et al., 2008; Li et al., 2013; Fanous et al., 2013)
3,4-methyl enedioxy methamphetamine (MDMA)		increases serotonin release and inhibits serotonin reuptake	sex-dependent changes in Arc protein in cortex and hippocampus	Llorente-Berzal et al. (2013)
methamphetamine (METH)	Desoxyn, Methedrine	induces release of dopamine, NE, 5-HT	increases *Arc* in parietal, orbital and medial PFC, striatum, and hippocampus	(Kodama et al., 1998; Fujiyama et al., 2003)
methylphenidate (MPH)	Ritalin, Ritalin SR, Ritalin LA, Aptensio XR, Concerta, Daytrana, Metadate, Metadate CD, Metadate ER, Methylin, Quillivant, QuilliChew ER	blocks reuptake of NE and dopamine	increases *Arc* in striatum and cingulate cortex	Chase et al. (2007)
morphine	AVINza, Kadian, Kadian ER, Morphabond, MS Contin, Oramorph SR, Roxanol, Roxanol-T	opioid analgesic	treatment increases *Arc* and Arc in striatum, and increases Arc in the NAc core; withdrawl increases *Arc* in central and basolateral amygdala	(Marie-Claire et al., 2004; Lucas et al., 2008; Lv et al., 2011)
nicotine		blocks nicotonic acetylcholine receptors	increases *Arc* in cingulate cortex, hippocampus, caudate, central amygdala, and bed nucleus of stria terminalis	Schmitt et al. (2008)
tetrahydrocannabinol (THC)		activates cannabinoid-1 receptors	sex-dependent changes in Arc protein in cortex and hippocampus	Llorente-Berzal et al. (2013)
**Drugs of Abuse in Combination with Other Compounds**				
BDNF and alcohol		neurotrophic factor, activates TrkB receptors	infusion of BDNF in central amygdala normalizes Arc levels after alcohol exposure	Pandey et al. (2008)
CTAP and METH		mu-opioid receptor antagonist	pretreatment of CTAP attenuates METH-induced increase in *Arc*	Horner et al. (2010)
6-hydroxydopamine (Oxidopamine) and cocaine		dopamine and noradrenergic neurotoxin	pretreatment of oxidopamine before cocaine administration suppresses increase in *Arc* in the striatum	(Fosnaugh et al., 1995; Fumagalli et al., 2006)
MK-801 and METH/heroin	Dizocilpine (MK-801)	NMDA receptor antagonist	pretreatment of MK-801 before METH or heroin administration abolishes increases in *Arc* or Arc in certain brain regions	(Kodama et al., 1998; Fujiyama et al., 2003; Li et al., 2013)
naloxone and morphine	Narcan, Evzio (naloxone)	opioid antagonist	naloxone-precipitated withdrawal from morphine maintains elevated levels of *Arc* in the frontal cortex	Ammon et al. (2003)
quinpirole and caffeine		dopamine 2 and 3 receptor agonist	pretreatment of quinpirole blocks caffeine-induced increase in *Arc* and Arc in striatonigral and striatopallidal neurons; direct administration of quinpirole has no change in *Arc*	(Dassesse et al., 1999; Fumagalli et al., 2009b)
reserpine and cocaine	Serpasil (Reserpine)	blocks reuptake of NE, dopamine, 5-HT	pretreatment of reserpine before cocaine administration suppresses increase in *Arc* in the striatum	(Fosnaugh et al., 1995; Fumagalli et al., 2006)
SCH- 23390 (halobenzazepine) and cocaine/METH/heroin/caffeine		dopamine receptor D1 antagonist	pretreatment of SCH- 23390 before cocaine, METH, heroin, or caffeine administration abolishes increase in *Arc* or Arc in certain brain regions	(Fosnaugh et al., 1995; Fumagalli et al., 2006; Kodama et al., 1998; Fujiyama et al., 2003; Li et al., 2013; Dassesse et al., 1999)

*Abbreviations:* 5-hydroxytryptamine (5-HT), acetylcholine (ACh), basolateral amygdala (BLA), brain-derived neurotrophic factor (BDNF), dorsal hippocampus (dHP), gamma-aminobutyric acid (GABA), H-_D_-Phe-Cys-Tyr-D-Trp-Arg-Thr-Pen-Thr-NH_2_ (CTAP), methamphetamine (METH), N-methyl-D-aspartate (NMDA), norepinephrine (NE), nucleus accumbens (NAc), orbitofrontal cortex (OFC), prefrontal cortex (PFC), tropomyosin receptor kinase B (TrkB).

Several drugs used in the management of Major Depressive Disorder (MDD) such as SSRIs and ketamine also affect Arc (Table 2). Repeated exposure to stress is a well-known risk factor for MDD (Wurtman, 2005). Acute and chronic stress are associated with upregulation of Arc and disruptions in glutamatergic transmission, and Arc levels are dysregulated in various models of MDD with depression-like behavior (Li et al., 2015; Penrod et al., 2019). Antidepressants alter Arc mainly in the cortex and striatum. Interestingly, their effect on Arc appears to be somewhat similar to their therapeutic course in humans, where little change is observed with acute treatments and more pronounced effects with a chronic course of treatment (Pei et al., 2003; Ferres-Coy et al., 2013; De Foubert et al., 2004; Alme et al., 2007), unlike the non-selective NMDAR antagonist ketamine, which is characterized by its rapid antidepressant effects, and induces Arc within hours (Li et al., 2010). The *Arc* gene has also been implicated in schizophrenia (Fromer et al., 2014; Purcell et al., 2014), with altered *Arc* mRNA detected in the PFC of schizophrenia patients (Guillozet-Bongaarts et al., 2014). Arc disruptions produce schizophrenia-like symptoms in mice through modulation of the dopaminergic system in the PFC and striatum (Manago et al., 2016). Both typical and atypical antipsychotic drugs alter Arc in the striatum, PFC and NAc through their actions on D2 receptors, suggesting that modulation of Arc may underlie some of their therapeutic effects (Robbins et al., 2008; Fumagalli et al., 2009b).

**Table 2:** List of psychotropic drugs, clinical names, general mechanism of action, and effect on Arc.

Psychotropic Drugs				
Drug	Clinical/Trade Name	General Mechanism	Effect on Arc	Reference
agomelatine	Valdoxan, Thymanax, Agoprex, Melitor, Vestin, Alodil, etc.	melatonergic MT_1_/MT_2_ receptor agonist and 5-HT_2C_ receptor antagonist	upregulates *Arc* in hippocampus	Calabrese et al. (2011)
amisulpride	Solian	high-affinity dopamine 2/3 receptor blocker	induces lower levels of *Arc* in medial striatum than haloperidol	de Bartolomeis et al. (2013)
clozapine	Clozaril, FazaClo ODT, Versacloz	binds serotonin, dopamine, and GABA_B_ receptors	decreases *Arc* in thalamus and hypothalamus; decreases Arc in medial PFC and cingulate cortex; increases Arc in NAc shell	(Robbins et al., 2008; Fumagalli et al., 2009b)
desipramine	Norpramin	monoamine oxidase inhibitor	chronic administration increases *Arc* in parietal, frontal, orbital, cingulate cortex, and CA1 hippocampus	( Pei et al., 2003)
1-(2,5-dimethoxy-4-iodophenyl)-2-aminopropane (DOI)		5-HT_2_ agonist	increases *Arc* in cortex and striatum	Pei et al. (2000)
duloxetine	Cymbalta	serotonin-norepinephrine reuptake inhibitor	chronic treatment induces *Arc* in frontal cortex	Molteni et al. (2008)
escitalopram	Cipralex, Lexapro	selective serotonin reuptake inhibitor	chronic treatment of escitalopram along with stimulation of MAPK signaling cascades restores *Arc* levels in the PFC and hippocampus in Flinders sensitive line rats	Eriksson et al. (2012)
haloperidol	Haldol, Haldol Decanoate, Haloperidol LA, Peridol	dopamine receptor D2 antagonist	increases *Arc* in striatum, *Arc* and Arc (acute treatment) in NAc, and Arc in caudate nucleus; decreases *Arc* in frontal cortex	(Fosnaugh et al., 1995; Fumagalli et al., 2006, 2009b; Robbins et al., 2008)
ketamine	Ketalar, LidoProfen	non-selective NMDA receptor antagonist, activates mTOR signaling pathway	increases Arc in PFC	Li et al. (2010)
L-tryptophan	Tryptan	increase 5-HT synthesis	no change in *Arc*	Pei et al. (2000)
olanzapine	Zyprexa, Zyprexa Relprevv, Zyprexa Zydis	blocks serotonin, dopamine, and muscarinic receptors	increases *Arc* in striatum, decreases *Arc* in frontal cortex	Fumagalli et al. (2009b)
paroxetine	Paxil, Brisdelle, Paxil CR, Pexeva	serotonin reuptake inhibitor; monoamine oxidase inhibitor	chronic administration increases *Arc* in parietal, frontal, orbital, cingulate cortex, and CA1 hippocampus	( Pei et al., 2003)
phencyclidine (PCP)	Sernyl, Sernylan	NMDA antagonist	alters *Arc* in medial PFC, orbitofrontal cortex, and NAc shell	(Thomsen et al., 2010; Nakahara et al., 2000)
spiperone	Spiropitan	dopamine receptor D2 antagonist	increases *Arc* in striatum	(Fosnaugh et al., 1995; Fumagalli et al., 2006)
thioridazine	Mellaril	inhibit Arc N-lobe binding, blocks dopamine 1/2 receptors, serotonin receptors, H1 receptors, and alpha-adrenergic receptors	inhibits TARPγ2 binding to Arc N-lobe	Zhang et al. (2015)
tranylcypromine	Parnate	monoamine oxidase inhibitor (MAOI)	chronic administration increases *Arc* in dentate gyrus, acute administration increases *Arc* in parietal cortex	(Pei et al., 2003)
trifluoperazine	Stelazine	inhibit Arc N-lobe binding, blocks dopamine 1/2 receptors	inhibits TARPγ2 binding to Arc N-lobe	Zhang et al. (2015)
venlafaxine	Effexor, Effexor XR	monoamine oxidase inhibitor	chronic administration increases *Arc* in parietal, frontal, orbital, cingulate cortex, and CA1 hippocampus	( Pei et al., 2003)
**Psychotropic Drugs in Combination with Other Compounds**				
caffeine and haloperidol			cotreatment with caffeine and haloperidol reduces increase in Arc in striatum caused by haloperidol treatment alone	de Bartolomeis et al. (2018)
ketanserin and tranylcypromine with L-tryptophan	Sufrexal (ketanserin)	5-HT_2_ receptor antagonist	administration of ketanserin attenuates increase in *Arc* in cortex and striatum from tranylcypromine and L-tryptophan administration	Pei et al. (2000)
NDA-299 and paroxetine		5-HT_1A_ receptor antagonist	administration of NDA-99 with paroxetine increases *Arc* in caudate putamen, frontal, parietal, and piriform cortices	(Tordera et al., 2003; Castro et al., 2003)
nicotine and haloperidol			cotreatment with nicotine and haloperidol increases Arc in cortex than haloperidol treatment alone	de Bartolomeis et al. (2018)
p-chlorophenylalanine and tranylcypromine with L-tryptophan	Fenclonine (p-chlorophenylalanine)	tryptophan hydroxylase inhibitor	administration of p-chlorophenylalanine attenuates increase in *Arc* in cortex and striatum from tranylcypromine and L-tryptophan administration	Pei et al. (2000)
tranylcypromine and L-tryptophan	Parnate (tranylcypromine)	monoamine oxidase inhibitor (MAOI)	combined administration of tranylcypromine with L-tryptophan increases *Arc* in striatum, orbital cortex, frontal cortex, and parietal cortex; decreases *Arc* in CA1 hippocampus	Pei et al. (2000)
WAY 100635 and paroxetine	Nefazodone (WAY 100635)	5-HT_1A_ receptor antagonist	administration of WAY 100635 with paroxetine increases *Arc* in caudate putamen, frontal, parietal, and piriform cortices	(Tordera et al., 2003; Castro et al., 2003)

*Abbreviations:* 5-hydroxytryptamine (5-HT), gamma-aminobutyric acid (GABA), histamine 1 (H1), mammalian target of rapamycin (mTOR), N-methyl-D-aspartate (NMDA), nucleus accumbens (NAc), prefrontal cortex (PFC), transmembrane AMPA receptor regulatory protein subunit γ 2 (TARPγ2).

Arc is modulated by BDNF which regulates neuronal survival and differentiation (Yin et al., 2002). BDNF has been extensively investigated as a potential therapeutic tool for spinal cord injuries (Awad et al., 2015), neurodegenerative diseases (Sampaio et al., 2017) and stem cell therapy (Pramanik et al., 2017) with unclear results in human studies (Zoladz and Pilc, 2010; Palasz et al., 2020). Furthermore, Arc is implicated in several neurodegenerative diseases including AD, where it is found to be dysregulated by Aβ oligomers (Lacor et al., 2004). Studies show abnormal levels of Arc in response to neuronal activation in various AD mouse models (Kerrigan and Randall, 2013; Morin et al., 2016) and in human postmortem AD brains (Wu et al., 2011). Arc binds to presenilin 1 (PSI), the catalytic subunit of the gamma-secretase complex, and disruption of the Arc-PS1 interaction prevents activity-dependent increases in soluble hAβ40 (Wu et al., 2011). AD has been characterized as a chronic inflammatory state (Kinney et al., 2018). Intriguingly, memantine, an NMDA antagonist widely used to treat symptoms of mild to moderate AD (Folch et al., 2018), reverses the disruptions of behaviorally-induced Arc in chronic inflammation, which appear to be mediated via NMDAR-related mechanisms, perhaps adding another layer to Arc's proposed neuronal functions in AD (Rosi et al., 2005, 2006). Additionally, Arc levels increase in response to inflammatory mediators such as IL-6 and NGF in dorsal root ganglia (Barragan-Iglesias et al., 2020) (Table 3).

**Table 3:** List of other compounds, clinical names, general mechanism of action, and effect on Arc.

Other Compounds				
Compound	Clinical/Trade Name	General Mechanism	Effect on Arc	Reference
AK-7		protein lysine deacetylase inhibitor	increases Arc by limiting its degradation in dentate gyrus and BDNF-treated primary cortical neuron cultures	Lalonde et al. (2017)
Brain-derived neurotrophic factor (BDNF)		neurotrophic factor, activates TrkB receptors	increases *Arc* and Arc in dentate gyrus; induces *Arc* in primary cortical neuronal cultures and hippocampal organotypic slices	(Ying et al., 2002; Rao et al., 2006)
CHIR 98014 (CH98)		GSK3α/β inhibitor	decreases Arc degradation	Gozdz et al. (2017)
1,3-dipropyl-8 cyclopentylxanthine (DPCPX)		adenosine 1 antagonist	increases *Arc* and Arc in striatonigral and striatopallidal neurons	Dassesse et al. (1999)
diamide		disulfide crosslinker	increases Arc (observed in HeLa cells)	Park et al. (2019)
eticlopride		dopamine receptor D2 antagonist	increases *Arc* in striatum	(Fosnaugh et al., 1995; Fumagalli et al., 2006)
FTY720	Fingolimod	functional sphingosine 1-phosphate receptor antagonist	decreases *Arc* in migratory dendritic cells	Ufer et al. (2016)
interleukin 6 (IL-6)		neuroinflammatory agent	increases Arc in dorsal root ganglion neurons	Barragan-Iglesias et al. (2020)
K252-alpha		tyrosine kinase inhibitor	pretreatment of K252-alpha before BDNF infusion inhibits BDNF-induced increase in Arc in synaptoneurosomes	Yin et al. (2002)
leupeptin		lysosomal inhibitor	leupeptin along with ammonium chloride inhibits autophagy pathway leading to Arc accumulation in hippocampal neurons	Yan et al. (2018)
lipopolysaccharide (LPS)		endotoxin that binds to TLR4 receptor, results in secretion of cytokines	induces *Arc* in dentate gyrus and CA3 hippocampus; no change in basal *Arc* or Arc translation/removal	(Rosi et al., 2005, 2006, 2009)
3-methyladenine (3-MA)		inhibits autophagosome formation	inhibits autophagy pathway leading to Arc accumulation in hippocampal neurons	Yan et al. (2018)
memantine	Namenda, Namenda XR	NMDA receptor antagonist	abolishes increase in *Arc* from LPS administration	(Rosi et al., 2006, 2009)
MG-132		proteasome inhibitor	decreases Arc degradation	(Mabb et al., 2014; Rao et al., 2006)
MK-801	Dizocilpine	NMDA receptor antagonist	pretreatment of MK-801 before BDNF infusion inhibits BDNF-induced increase in Arc in synaptoneurosomes	Yin et al. (2002)
nerve growth factor (NGF)		regulates cell growth and survival, released by mast cells in periphery	increases Arc in dorsal root ganglion neurons	Barragan-Iglesias et al. (2020)
oxamflatin		protein lysine deacetylase inhibitor	increases Arc by limiting its degradation in BDNF-treated primary cortical neuron cultures	Lalonde et al. (2017)
p-chloroamphetamine		5-HT releasing agent	increases *Arc* in cortex and striatum	Pei et al. (2000)
raclopride		dopamine 2 receptor antagonist	increases *Arc* in striatum, decreases *Arc* in frontal cortex	Fumagalli et al. (2009b)
Ro25-6981		NR2B antagonist, activates mTOR pathway	increases Arc in PFC	Li et al. (2010)
S35966A		dual α_2_-adrenoceptor antagonist and 5-HT-noradrenaline reuptake inhibitor	increases *Arc* in cortex and hippocampus	Serres et al. (2012)
sodium arsenite		binds to telomeric sequences to induce apoptosis	increases Arc (observed in HeLa cells)	Park et al. (2019)
U0126		mitogen-activated protein kinase inhibitor	abolishes increase in *Arc* observed from BDNF infusion	Ying et al. (2002)

*Abbreviations:* 5-hydroxytryptamine (5-HT), brain-derived neurotrophic factor (BDNF), glycogen synthase kinase 3α/β (GSK3α/β), lipopolysaccharide (LPS), mammalian target of rapamycin (mTOR), N-methyl-D-aspartate (NMDA), N-methyl-D-aspartate receptor subtype 2B (NR2B), prefrontal cortex (PFC), toll-like receptor 4 (TLR4), tropomyosin receptor kinase B (TrkB).

Taken together, these studies suggest that reversing abnormalities in Arc expression and function might be a key therapeutic strategy for various disorders that disrupt cognitive functions. Arc is pivotal to memory, learning and cognitive processing where Arc knock-out mouse models display behavioral phenotypes related to schizophrenia (Manago et al., 2016). To this end, Lalonde et al. performed a chemogenomic screen in search for small molecules capable of modulating Arc levels and function through BDNF signaling revealing that the majority of the drugs considered as “Arc suppressers” were drugs with antipsychotic or antidepressant activity, while the majority of “Arc potentiators” had neuroprotective and/or nootropic properties (Lalonde et al., 2017). It remains unclear whether modulating Arc in itself is sufficient to achieve the full therapeutic effects of these drugs. To the extent of our knowledge, only the first-generation anti-psychotics thioridazine and trifluoperazine can directly bind Arc and hold promise for pharmacological use to interfere with Arc binding partners (Zhang et al., 2015). Additionally, while injecting drugs that can modulate Arc into target brain areas is feasible in animal models, this remains a challenge in humans to avoid side-effects that might result from undesired widespread modulation of Arc in the brain. Addressing these challenges is key to the potential of Arc as a therapeutic target.

## Funding

This work was supported by a 10.13039/100009670NARSAD Young Investigator Grant from the 10.13039/100000874Brain & Behavior Research Foundation Research Partners Program (P&S Fund Investigator) and a 10.13039/100001391Whitehall Foundation (Grant 2017-05-35) to A.M.M. D.W.Y. was supported by a 10.13039/100008545Georgia State University Neurogenomics 2CI Fellowship. N.S. was supported by the Brains & Behavior summer scholars program and a Goldwater Scholarship.

## CRediT authorship contribution statement

**Dina W. Yakout:** Conceptualization, Writing - original draft, Writing - review & editing. **Nitheyaa Shree:** Writing - original draft, Writing - review & editing. **Angela M. Mabb:** Conceptualization, Supervision, Writing - original draft, Writing - review & editing.

## Declaration of competing interest

The authors declare that they have no known competing financial interests or personal relationships that could have appeared to influence the work reported in this paper.
